# Electrocardiographic Characteristics of Ventricular Arrhythmia Originating from the Left Coronary Cusp

**DOI:** 10.1155/2011/935951

**Published:** 2011-11-15

**Authors:** Antoine Kossaify, Nadir Saoudi, Sami Succar

**Affiliations:** ^1^Electrophysiology Unit, Cardiology Division, CHU-NDS/USEK, Byblos 00003, Lebanon; ^2^Electrophysiology Unit, Cardiology Division, CH Princess Grace, Monaco 98007, Monaco

## Abstract

Aortic cusps originating arrhythmias are rare; they have special electrocardiogram features that help to locate the site of origin. We report on a 20-year-old male patient without structural heart disease presenting with accelerated idioventricular rhythm; electrocardiogram analysis was typical of left coronary cusp origin.

## 1. Introduction

Outflow tract ventricular arrhythmias (VAs) usually have a benign course when occurring in the setting of normal structural heart. Left coronary cusp (LCC) originating arrhythmias have special electrocardiogram (EKG) characteristics; these are well established in the literature [1]; recognition of these characteristics is essential for accurate diagnosis and management.

## 2. Case Presentation

We report on a 20-year-old male patient presenting with palpitations; his EKG (Figure S1, which is available online at doi: 10.1155/2011/935951) showed accelerated idioventricular rhythm with EKG characteristics compatible with a LCC origin (Figure S2). The patient had no relevant past medical history; physical examination was normal, cardiac echogram showed no structural heart disease, and laboratory parameters including cardiac markers were normal. Reversion to sinus rhythm occurred with a 75 mg bolus of Xylocaine. Given the refusal of any electrophysiological procedure (diagnostic or therapeutic), the patient was discharged home on day 3 with verapamil. Holter and stress test on days 10 and 30 after discharge showed no VA and were normal.

## 3. Discussion

Supravalvular left ventricular outflow tract arrhythmias originate usually from the aortic sinus of valsalva (aortic cusps) [2]. VA originating from the right or left ventricular outflow tracts (RVOT and LVOT) can be resembling on surface EKG because these two structures are anatomically adjacent [3]. Both LVOT-VA and RVOT-VA exhibit right axis; RVOT-VA usually displays a typical left bundle branch block pattern without (r) wave in V1 because the activation pattern is typically from the right to the left ventricle [1]. 

LCC-VA is known to be the most common form of coronary cusp arrhythmia; it exhibits an (r) wave in V1 [1] (so-called atypical left bundle branch block pattern) because the activation vector is directed from posterior to anterior and from the left to the right (LCC is located posteriorly). Right coronary cusp and non-coronary cusp VAs do not exhibit an (r) wave in V1 [1] (these two anatomical structures are located more anteriorly compared to LCC) (Figure S2).

LVOT-VA displays earlier precordial transition zones (median V3 versus V5) compared to RVOT-VA [2]; this feature is due to earlier left to right activation pattern in case of LVOT-VA resulting in an earlier (r) wave consequently in precordial leads [3].

The absence of (S) wave in left precordial leads is also typical of left VA originating from coronary cusps, in contrary to VA originating in the left ventricular myocardium resulting in late right ventricular activation and typical right bundle branch pattern with prominent (S) wave in V5-V6 [4].

The presence of rS pattern in D1 [5] is also more prominent in LCC-VA whereas it is less marked or even absent in right coronary or non-coronary cusp; this is due to late activation of the right ventricle in case of LCC-VA because LCC is located more to the left and more posterior compared to the two other cusps.

When invasive electrophysiological studies could not be performed, surface EKG becomes of utmost importance to diagnose this type of VAs and accordingly to differentiate it from other VAs, especially non-outflow tract VA which have a more critical prognosis if they are not treated adequately [6]. Outflow tract VA have a favorable prognosis in the majority of patients, and the risk of sudden cardiac death is low when no structural heart disease is documented [6]. When medical therapy is not sufficient to control VA, transcatheter ablation procedures may offer a definite therapy to most patients.

## 4. Conclusion

LCC-VAs have special EKG characteristics and accordingly they can be diagnosed with sufficient accuracy based on surface EKG.

##  Limitations 

in this presented case, there was no invasive electrophysiological procedure performed due to patient denial and so intracavitary mapping of the arrhythmia is lacking.

##  Conflict of Interests 

The authors declare that there is no conflict of interest.

##  Consent 

According to our institutional Ethics Committee, a written consent was obtained from the patient before proceeding with this paper.

## Supplementary Material

Figure 1. Legend: EKG showing accelerated idioventricular rhythm with EKG characteristics compatible with a left coronary cusp origin.
Figure 2. Legend: EKG (s) of different types of Coronary cusps ventricular arrhythmias. (Courtesy of Yamada et al. (2008)).
