# Supplementary figures and images for: Electrocardiographic Characteristics of Ventricular Arrhythmia Originating from the Left Coronary Cusp
Source: Case Rep Med. 2011 Nov 15;2011:935951. doi: 10.1155/2011/935951 (PMC3227424; doi:10.1155/2011/935951)

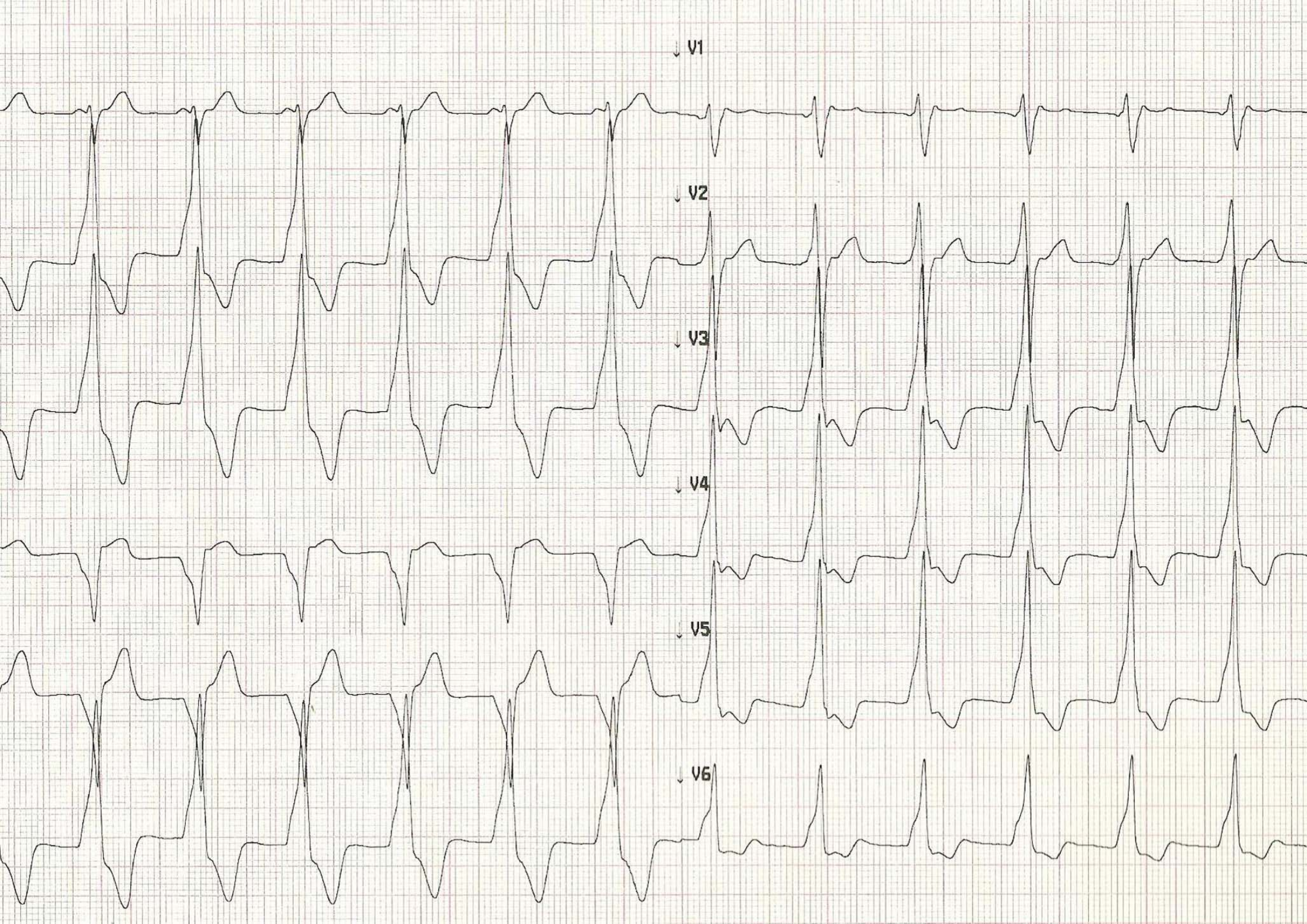

Supplement: Supplementary file 1 — Figure 1. Legend: EKG showing accelerated idioventricular rhythm with EKG characteristics compatible with a left coronary cusp origin. Figure 2. Legend: EKG (s) of different types of Coronary cusps ventricular arrhythmias. (Courtesy of Yamada et al. (2008)). [file 935951.f1.pdf]

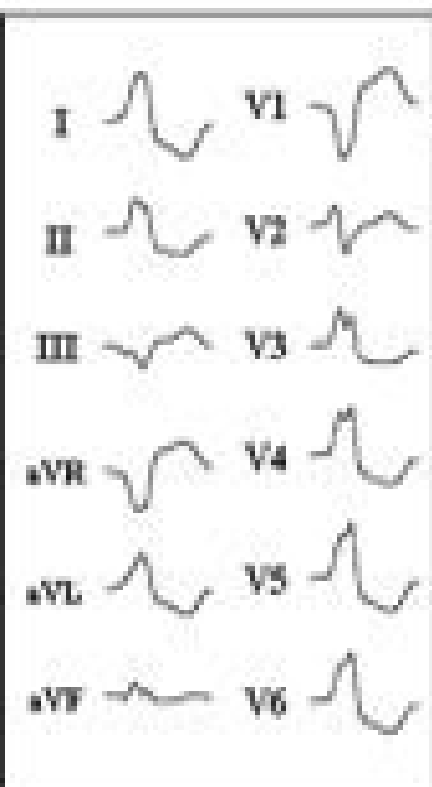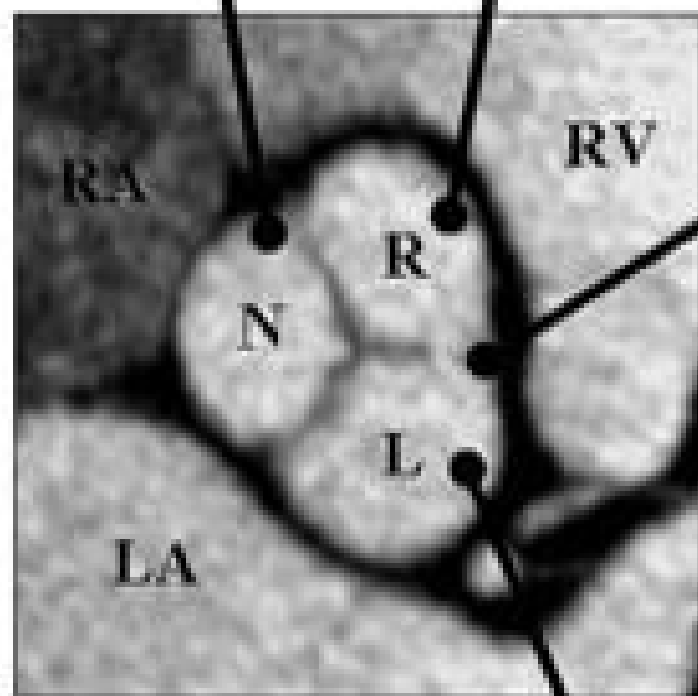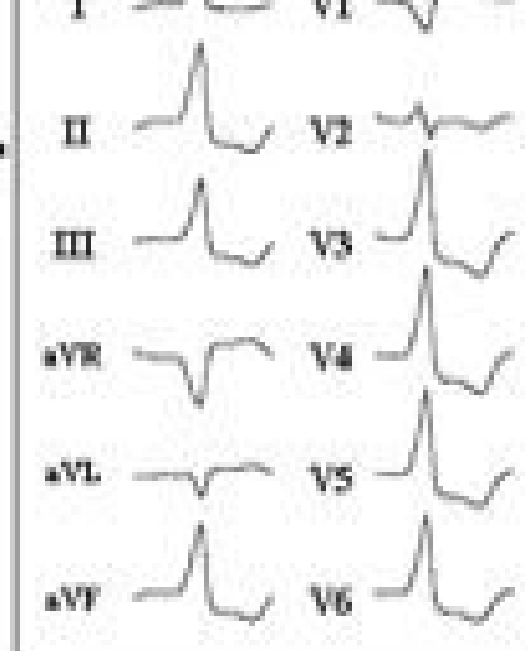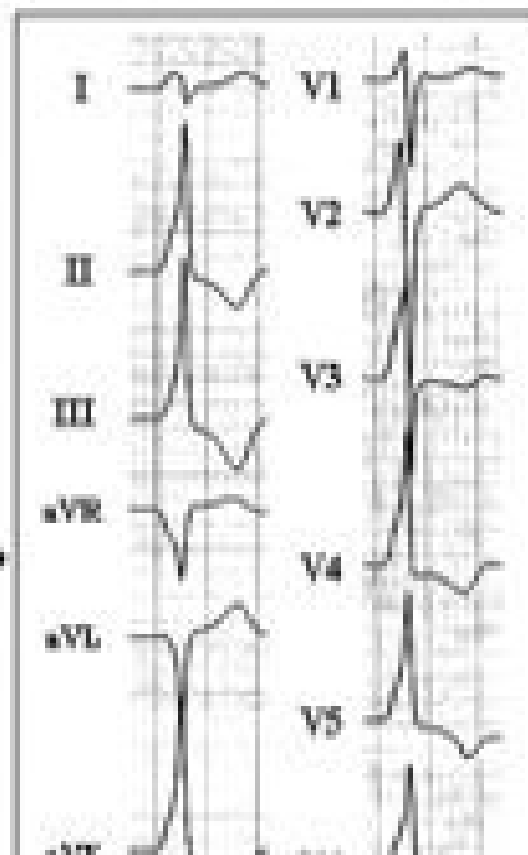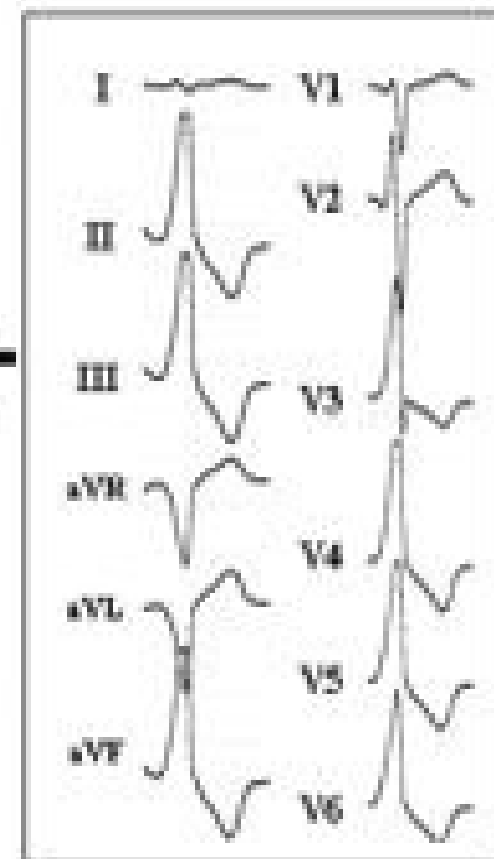

1 mV  
200 ms

Supplement: Supplementary file 2 [file 935951.f2.pdf]
